# Is It Possible for Children in Duchenne Muscular Dystrophy to Preserve Cardiac Function with Medical Assistance?

**DOI:** 10.3390/children7110249

**Published:** 2020-11-22

**Authors:** Han Geul Kim, Lucy Youngmin Eun, Han Ki Park

**Affiliations:** 1 Division of Pediatric Cardiology, Department of Pediatrics, Yonsei University College of Medicine, Seoul 03722, Korea; khg7464@yuhs.ac; 2 Division of Cardiovascular Surgery, Department of Thoracic and Cardiovascular Surgery, Yonsei University College of Medicine, Seoul 03722, Korea

**Keywords:** Duchenne muscular dystrophy, children, cardiomyopathy, heart failure, echocardiography, medication

## Abstract

In patients with Duchenne muscular dystrophy (DMD), death secondary to cardiac or respiratory failure typically occurs in the second or third decade without treatment. Although cardiac dysfunction is treated with standard heart-failure strategies, it remains insufficient in DMD children. The purpose of this study was to evaluate the efficiency of cardiac medication and noninvasive ventilator support in DMD cardiomyopathy children with analyzing echocardiographic data. Forty-eight DMD children patients were included and divided into 2 groups by left ventricular (LV) ejection fraction (EF) at the time of initial treatment. Group 1: LV EF ≥ 45% and Group 2: LV EF < 45%. *p*-values were calculated using a Linear mixed model to estimate the association between cardiac medications and echocardiographic measurements. Before and after cardiac medications, the change values were significantly different in interventricular septal thickness at end diastole (IVSd), interventricular septal thickness at end systole (IVSs), left ventricular internal diameter end systole (LVIDs), left ventricular posterior wall thickness end diastole (LVPWd), ejection fraction (EF), fractional shortening (FS), deceleration time (DT), DT slope, Lat A’ and Lat E/E’ (*p* < 0.05). Group 2 patients revealed to take more kinds of cardiac medications than Group 1 (*p* < 0.05) including ACEIs, beta-blocker, and inotropics, then LV EF was better preserved in Group 2 than Group 1. It is certainly helpful to take individualized medical combination therapy including inotropic agents for cardiomyopathy in DMD children patients with EF < 45%.

## 1. Introduction

Duchenne muscular dystrophy (DMD), an X-linked disorder due to lack of dystrophin, is characterized by progressive muscle weakness and myocardial dysfunction [1]. DMD is typically diagnosed between the ages of 3 and 7 years and is characterized by progressive skeletal muscle weakness with loss of ambulation between the ages of 7 and 13 years [2]. Without treatment, death secondary to cardiac or respiratory failure typically occurs in the second or third decade in DMD. Respiratory care of DMD patients has improved as a result of the development of supportive equipment and techniques [3]. Advances in the respiratory care of patients with DMD have improved their prognosis. Nocturnal home ventilators and mechanically assisted coughing lead to improved survival of patients with DMD [4]. The American Thoracic Society has published a statement regarding the respiratory care of patients with DMD, including evaluation and management (i.e., respiratory muscle training, mechanical ventilation, corticosteroids, and end of-life care) [5]. These improvements in respiratory care make cardiac complications the leading cause of death in patients with DMD [6,7,8,9,10]. Consequently, dilated cardiomyopathy and depressed cardiac function are increasing as the major cause of death.

Evidence of myocardial involvement begins with minor electrocardiographic abnormalities [11]. Then cardiac involvement evolves to cardiomyopathy with dilatation of the cardiac chambers and depression of left ventricular ejection fraction due to widespread fibrosis. Progression of cardiomyopathy is the major cause of mortality. Cardiomyopathy can occur at any age but often occurs around 14–15 years [12,13].

Although cardiac dysfunction is treated with standard heart-failure strategies, it remains insufficient in DMD children. The treatment paradigms have been individually based and relied on evidence acquired from other patient populations [2]. There are some reports that support the efficacy of angiotensin-converting enzyme inhibitors (ACEIs) on left ventricular function and mortality of DMD patients [11]. In addition to ACEIs, angiotensin receptor blockers, beta-blockers, or aldosterone antagonists are often used for management of Duchenne cardiomyopathy and show improvements in cardiac function. Oral corticosteroid treatment can delay the onset of Duchenne cardiomyopathy [14]. However, except for ACEIs, there are no reports on the efficacy of other medications.

Baseline assessment of cardiac function is needed for patients with DMD at diagnosis, and annual cardiac assessment is recommended for patients with DMD older than 10 years. Poor treatment outcomes have been noticed in patients with DMD who fail to see a cardiologist after the onset of clinical symptoms of heart failure [2,11,12,13].

In our hospital, we treated DMD cardiomyopathy patients with cardiac medication and respiratory care. After the start of treatment, we could see the improvement of patients’ early symptoms of heart failure like poor oral intake, night sweat, chest discomfort, fatigue, palpitation or sleep disturbance etc. Based on this, the purpose of this study was to evaluate the efficiency of cardiac medication in DMD cardiomyopathy patients with analyzing echocardiographic data to preserve the cardiac function.

## 2. Experimental Section

### 2.1. Patient Enrollment and Data Measurement

**Figure 1:**
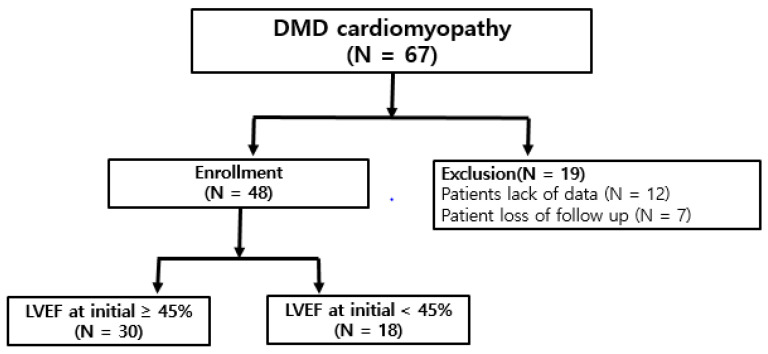
Sixty-seven DMD (Duchenne muscular dystrophy) cardiomyopathy patients’ data were collected. Nineteen patients were excluded due to the lack of data or loss of follow up. Forty-eight DMD patients were divided into 2 groups of left ventricle ejection fraction (LVEF) at initial treatment over 45% (Group 1) and under 45% (Group 2). N; number.

We collected sixty-seven DMD cardiomyopathy patient’s data in Gangnam Severance Hospital, Korea, from January 2014 to December 2018. Among them, forty-eight patients were enrolled in this study, twelve patients were excluded due to the lack of data and seven patients were excluded due to the loss of follow up (Figure 1).

We retrospectively reviewed the medical record of their cardiac medications (Beta-blocker, ACE inhibitor, Diuretics, Inotropics, Aspirin etc.), echocardiographic data, demographic data and non-invasive ventilator apply time per day.

For each patient diagnosed with DMD cardiomyopathy, we performed echocardiography at least one time before treatment and at least one time after treatment. We acquired conventional and advanced echocardiographic data for analyzing myocardial function. All conventional echocardiographic measurements and advanced myocardial imaging studies were obtained in all enrolled patients with DMD using a Siemens model ACUSON SC2000 (Siemens Medical Solutions USA, Inc., Mountain View, California, USA). Echocardiographic examinations were conducted according to the recommendations of the American Society of Echocardiography, and based on the guidelines and Standards for Performance of a Pediatric Echocardiogram: A Report from the Task Force of the Pediatric Council of the American Society of Echocardiography [15]. The parameters included in this study are listed: Interventricular septal thickness at end diastole (IVSd), Interventricular septal thickness at end systole (IVSs), Left ventricular internal diameter end diastole (LVIDd), Left ventricular internal diameter end systole (LVIDs), Left ventricular posterior wall thickness end diastole (LVPWd), Left ventricular posterior wall thickness end systole (LVPWs), End-diastolic volume (EDV), End-systolic volume (ESV), Ejection fraction (EF), Stroke volume (SV) and Fractional shortening (FS). Doppler measurement data included of mitral E, mitral A, E/A, Deceleration time (DT), DT slope, tissue Doppler Septal (Sep) E’, Sep A’, Sep S’, Sep E/E’, Lateral (Lat) E’, Lat A’, Lat S’ and Lat E/E’.

### 2.2. Study Design and Statistical Analysis

The change values of echocardiographic functional measurements were calculated subtracting the initial echocardiographic data from the last echocardiographic data and we analyzed these change values with linear mixed model. Then, we evaluated the relationship between significant change values and cardiac medication with respiratory care by linear regression. To analyze the treatment outcome between the two groups which is divided based upon LV EF (left ventricular ejection fraction), we evaluated relationship between the change values of echocardiographic parameters and cardiac medication and respiratory care with linear mixed interaction test. The *p* values were set using a linear mixed model to evaluate the change of each echocardiographic data with cardiac medication over time, and estimate the interaction between the change values of EF in each group. For this analysis, variables with *p* < 0.05 on the unadjusted analysis were entered.

Statistical analyses were performed using SAS ver. 9.3 (SAS Inc., Cary, NC, USA). The *p*-values less than 0.05 were considered statistically significant.

### 2.3. Ethics Statement

This study was approved by the Yonsei University College of Medicine Institutional Review Board and the Research Ethics Committee of Severance Hospital (study approval number: 2020-0697-001). All research was performed in accordance with relevant guidelines and regulations. The requirement for written informed consent was waived by the Institutional Review Board due to the retrospective study design.

## 3. Results

We reviewed 48 DMD patients and divided into two groups based on their ejection fraction at initial treatment. The demographic characteristics of patients with DMD are shown in Table 1. All patients were male. Age at first medication in Group 2 (17.11 ± 2.30 years) was older than in Group 1 (14.3 ± 5.77 years) (*p* < 0.05). For medications, Group 2 (4.6 ± 1.58) presented to take more kinds of medications than Group 1 (3.07 ± 1.26) (*p* < 0.05). But onset age of ACE inhibitor was similar between two groups. For height and weight of the patients, Group 1 (143.663 ± 4.606 cm) was smaller than Group 2 (158.556 ± 1.7827 cm) (*p* < 0.05) and Group 2 (45.7167 ± 2.822 kg) weighed more than Group 1 (34.7067± 2.5223 kg) (*p* < 0.05). Ventilator apply time per day and initial end-tidal CO_2_ (EtCO_2_) was not significantly different between the two groups. Initial EtCO_2_ was not statistically different between patients treated with ventilator and patients without ventilator. The follow-up duration was similar between two groups from 6 months to 4 years. They have been in treatment at least two years, and at most 6 years. As the time of their first clinic visit is individually different, the start of treatment is various for each patient.

The change values of systolic and diastolic echocardiographic data and the mean values of echocardiography which were taken before and after treatment are shown in Table 2. The change values were significantly different in IVSd, IVSs, LVIDs, LVPWd, EF, FS, DT, DT slope, Lat A’ and Lat E/E’ (*p* < 0.05). For the detail, IVSd became thinner by 0.07 cm and IVSs by 0.06 cm per year. LVPWd became thinner by 0.05 cm per year. LVIDs enlarged 0.13 cm per year. LV EF decreased 2.68%, FS 1.72% per year. With the Doppler data, DT decreased 7.3 ms, DT slope increased 0.6 m/s^2^ per year. Lat A’ became shorter by 0.41 cm/s, Lateral E/E’ changed by 0.57 per year (Table 2).

We assessed the impact of cardiac medication on these echocardiographic data with linear regression analysis (Table 3). Most of the parameters are not statistically associated with the medications, except the relevance between IVSd and diuretics. The use of diuretics accelerated the thinning of IVSd by 0.06 cm per year (*p* < 0.05).

The change values of the EF between Group 1 and Group 2 are shown in Table 4. In Group 1, EF decreased with 3.62% per year of statistical significance, while in Group 2, EF became decreased with 0.58% per year (*p* < 0.05). With the result in Table 1, we compared medications in two groups to evaluate which medication related LV EF most. Obviously, inotropic agents were more taken in Group 2 patients (Table 5).

**Figure 2:**
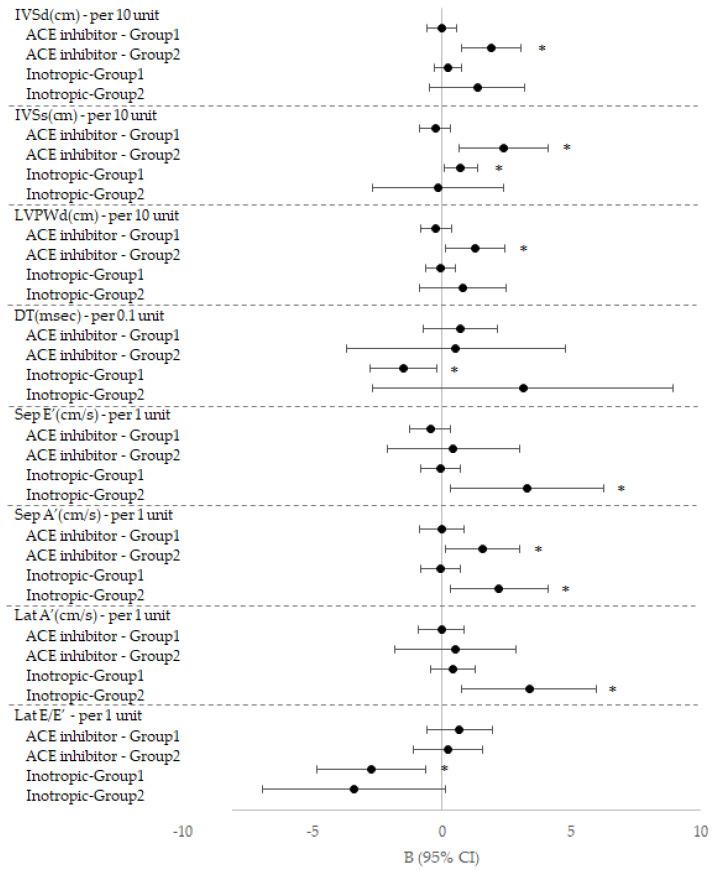
The effect of ACE inhibitor and inotropic agents on echocardiographic measurements in each group. IVSd: Interventricular septal end diastole; IVSs: Interventricular septal end systole, LVIDd: Left ventricular internal diameter end diastole; LVIDs: Left ventricular internal diameter end systole; LVPWd: Left ventricular posterior wall end diastole; LVPWs: Left ventricular posterior wall end systole; EDV: End-diastolic volume; ESV: End-systolic volume; EF: Ejection fraction; SV: Stroke volume; FS: Fractional shortening; DT: Deceleration time; E: peak early inflow velocity; A: peak late inflow velocity; E’: peak early diastolic velocity; A’: peak late diastolic velocity; S’: peak systolic velocity; *: *p*-value < 0.05.

To compare the effect of treatment, we analyzed relationship between the change values of echocardiographic data and cardiac medication & respiratory care in each group (Table 6). Taking ACE inhibitor in Group 2 was related to increase in IVSd, IVSs and LVPWd (*p* < 0.05), and also related to decrease in Sep S’ and Lat E/E’ and increase in Sep A’ (*p* < 0.05). Taking inotropic in Group 2 was related to increase in Sep E’ and Lat A’ (*p* < 0.05). Taking inotropic agents in Group 1 was related to increase in IVSs and decrease in DT (*p* < 0.05). The effect of these two medications, ACE inhibitor and inotropic agent in each group was demonstrated as a graph by Forest plot (Figure 2). Taking beta-blocker in Group 2 was related to decrease in EDV, EF, SV and FS (*p* < 0.05). Taking aspirin in Group 1 related to increase in IVSs and taking aspirin in Group 2 was related to decrease in SV (*p* < 0.05). Taking diuretics in Group 2 was related to decrease in Sep E/E’ (*p* < 0.05). Ventilator use in Group 2 was related to increase in mitral E (*p* < 0.05) (Table 6).

## 4. Discussion

DMD is an inherited X-linked disease with a 1/3000 male birth incidence. The disease follows a predictable clinical course marked by progressive skeletal muscle weakness. Death occurs in early adulthood secondary to respiratory or cardiac failure [16]. Cardiac involvement begins as minor electrocardiographic abnormalities and evolves toward cardiomyopathy with dilatation of the cardiac chambers and depressed LV EF due to widespread fibrosis; it accounts for up to 40% of deaths [17]. Cardiac management has been challenging because the New York Heart Association classification of heart failure relies on reduced exercise tolerance, a feature that in DMD arises from skeletal muscle and cardiac disease combined. In DMD children, the signs and symptoms of heart failure in the non-ambulatory individual are frequently subtle and overlooked. The patients with lower LV EF did not always demonstrate definite symptoms or signs of heart failure with chronic adjustment. Relatively, they had been in subtle difficulty with chest tightness, discomfort, or tachypnea. As DMD children might have been adjusted to their gradual progress of cardiac dysfunction, their clinical presentation of heart failure is probably masked or unusual compared with other patients with heart failure. Once or twice per year, they were regularly hospitalized for checking the spontaneous respiration or assisted ventilation with CO_2_ status, in addition to cardiac functional evaluation by echocardiography. For the patients with EF < 50%, we added low dosage of oral inotropic agents by combination of dopamine with dobutamine for stabilizing the vital sign of blood pressure and heart rate for optimal circulation. Among the enrolled patients, nobody was related implantable cardioverter defibrillator (ICD) or pacemaker implantation. Perhaps a proactive strategy of early diagnosis and treatment for cardiomyopathy in DMD is essential to maximize duration and quality of life [18].

In this study, we can notice that all the echocardiographic measurements demonstrated alteration over time even with or without statistical significances (Table 2). Myocardial wall thickness of IVSd, IVSs and LVPWd became thinner, systolic functional measurement of EF and FS decreased with ongoing cardiac dysfunction in DMD patients with aging (Table 2). We investigated to find the relation between cardiac medications and all the change values of echocardiographic parameters, and revealed the relationship (Table 3). Interventricular septal thickness at diastole implies the relaxation status of the radial direction of myofibril from both left ventricle (LV) and right ventricle (RV). While heart failure proceeds in DMD cardiomyopathy, myocardial thinning might develop at the interventricular septum, then each free wall of LV and RV in order. As diuretic is one of the medical treatment regimens for heart failure, for ongoing heart failure status in DMD children, the initial treatment protocol should include diuretics, ACE inhibitor, and inotropic agents, which helped the myocardial protection. However, beta-blocker was not contributed to maintain cardiac function. 

Moreover, we divided patients into two group based on EF which represents systolic function (Table 4) [19]. In Group 1, EF decreased by 3.615% annually, while in Group 2, EF decreased by 0.582% in similar respiratory assist situation (*p* < 0.05). The difference should be elucidated why the lower LV EF group demonstrated better preservation of cardiac function. In Table 5, it is noticeable that Group 2 patients take more numbers of medications (*p* = 0.001), especially including inotropic agents for stabilizing their blood pressure and heart rate at 88.9% in Group 2 (*p* = 0.0009).

It is well known that ACE inhibitor and beta-blocker are helpful for the treatment of heart failure. Furthermore, ACE inhibitor is recognized to decrease mortality in 10-year follow-up [20]. Similarly, in our study, there was significant positive correlation between ACE inhibitor and the echocardiographic parameters (Table 6). As Figure 2 demonstrated, especially in Group 2, by taking ACE inhibitor, IVSs, IVSd and LVPWd were more preserved and improved systolic and diastolic myocardial function of the measurement of Sep S’, Lat E/E’. In addition, taking inotropic agents showed statistical significance within similar reasonable ranges of Sep E’, Lat A’, DT, Lat S’, which implied diastolic and systolic functional preservation. While ACE inhibitor helped to protect myocardial wall thickness from thinning, but, diminished myocardial wall movement, inotropic agents effected to enhance systolic myocardial wall velocities by tissue Doppler measurements (Figure 1). Accordingly, ACE inhibitor with inotropic agents would benefit for cardiac functional preservation, especially in patients with lower LV EF.

In contrast, taking beta-blocker in Group 2 seemed to be associated deterioration of systolic function (EF, SV, EDV and FS). Based on this result, we could not expect favorable outcome with taking beta-blocker in patients with low EF. Despite, it is opposite result of conventional treatment, further research on this aspect should be necessary in the future.

LV EF is the most popular cardiac functional prognostic measurement in DMD; however, recent studies pointed out its low sensitivity to detect early cardiac involvement [11]. In addition, it is not common to use inotropic agents in DMD children whose EF is maintained within normal range. Goudot et al. reported that DMD patients without the presence of inotropic reserve (defined as an increase in LV EF >10% during dobutamine infusion) showed more significant decline of LV EF than DMD patients with the presence of inotropic reserve (*p* = 0.031 for difference in trend between groups). Moreover, they also reported an assessment of inotropic reserve may offer a sensitive approach for progression of cardiovascular disease in DMD children patients [17]. Based on inotropic reserve, to investigate the effect of inotropics on cardiac function in DMD cardiomyopathy, would be helpful to provide a better guideline for the treatment of DMD children. In this study, various medical treatment including inotropic agents revealed to benefit, not only for the stable blood pressure and heart rate, but also for preservation of myocardial function with ongoing heart failure in DMD children’s cardiomyopathy. Inotropic agents demonstrated to help to augmentation of Sep E’, Sep A’ and Lat S’ velocities in patients with LV EF < 45% (Table 5), which implied to sustain myocardial velocities.

The limitation of this study is that this is a single-center retrospective study and the numbers of patients was relatively small due to DMD disease’s rarity. We did not investigate the steroid treatment in this study [21]. The eplerenone, a new medication which is reported mineralocorticoid receptor antagonists in heart failure with preserved ejection fraction (HFpEF), we should investigate the effect for the DMD cardiomyopathy in the future [8].

Observation for the relationship between medications and echocardiographic parameters in this study was relatively short-term follow up for complete explanation. Further long-term research should be preceded for the fruitful results.

## 5. Conclusions

This study is very important for the cardiomyopathy in DMD children to preserve their myocardial function with medical treatment including ACE inhibitors, beta-blocker, diuretics, and inotropic agents. Especially for the patients with lower LV EF, ACE inhibitors with inotropic agent combination therapy might be beneficial to preserve cardiac function. Further research should be needed for beta-blocker and diuretics.

We suggest it is supportive to take delicate individualized combination therapy including ACE inhibitor and inotropic agents for cardiomyopathy in DMD patients with EF < 45%, rather than usual heart failure therapy.

## Figures and Tables

**Table 1:** Demographic characteristics of patients with Duchenne muscular dystrophy.

Characteristics	Mean ± SD	Minimum Value	Maximum Value	*p*-Value
Age at first medication (year) (*N* = 48)	15.35 ± 4.93	2	22	0.022
Group 1 (*N* = 30)	14.30 ± 5.77	2	21	-
Group 2 (*N* = 18)	17.11 ± 2.30	14	22	-
Age of the ACEi onset (year) (*N* = 37)	16.03 ± 3.97	2	22	0.257
Group 1 (*N* = 22)	15.41 ± 0.10	2	21	-
Group 2 (*N* = 15)	16.93 ± 0.64	14	22	-
Height (cm) (*N* = 48)	149.25 ± 21.60	75.2	176	0.005
Group 1 (*N* = 30)	143.67 ± 4.61	75.2	176	-
Group 2 (*N* = 18)	158.56 ± 1.78	145	170	-
Weight (kg) (*N* = 48)	38.84 ± 14.09	9.4	65.55	0.007
Group 1 (*N* = 30)	34.71 ± 2.52	9.4	65.55	-
Group 2 (*N* = 18)	45.72 ± 2.82	22	65	-
Number of medication (*N* = 48)	3.65 ± 1.56	0	7	0.001
Group 1 (*N* = 30)	3.07 ± 1.26	1	6	-
Group 2 (*N* = 18)	4.60 ± 1.58	0	7	-
Ventilator apply time per day (hour) (*N* = 48)	4.42 ± 4.99	0	19	0.977
Group 1 (*N* = 30)	4.43 ± 4.94	0	19	-
Group 2 (*N* = 18)	4.38 ± 5.23	0	17	-
Initial EtCO_2_ (*N* = 38)	36.76 ± 6.29	24	53	0.611
Group 1 (*N* = 24)	37.17 ± 6.40	24	53	-
Group 2 (*N* = 14)	36.07 ± 6.27	25	51	-
Initial EtCO_2_ (*N* = 38)	36.76 ± 6.29	24	53	0.09
Ventilator (*N* = 24)	38.08 ± 6.84	24	53	-
Non-ventilator (*N* = 14)	34.05 ± 4.59	25	51	-
Duration of follow up (year) (*N* = 48)	1.57 ± 0.63	0.5	3.75	0.14
Group 1 (*N* = 30)	1.66 ± 0.72	0.5	3.75	-
Group 2 (*N* = 18)	1.41 ± 0.42	1	2.33	-

*N*: number; Group 1: Ejection fraction at initial treatment over 45%; Group 2: Ejection fraction at initial treatment under 45%; EF: Ejection fraction; EtCO_2_: End-tidal CO_2_; ACEi: Angiotensin-converting enzyme inhibitor.

**Table 2:** The change values of echocardiographic data over time and the mean values of echocardiography before and after treatment.

Variable	Treatment		Linear Mixed Model		Variable	Treatment		Linear Mixed Model	
	Before (S.E)	After (S.E)	B (S.E)	*p*-Value		Before (S.E)	After (S.E)	B (S.E)	*p*-Value
IVSd (cm)	0.720 (0.194)	0.588 (0.118)	−0.069 (0.014)	<0.0001	Mitral E (cm/s)	80.511 (19.320)	83.222 (15.750)	1.113 (1.329)	0.4065
IVSs (cm)	0.938 (0.221)	0.805 (0.130)	−0.059 (0.017)	0.0008	Mitral A (cm/s)	46.956 (13.270)	51.689 (14.550)	1.446 (1.383)	0.3012
LVIDd (cm)	4.460 (0.988)	4.572 (1.018)	0.086 (0.046)	0.0688	E/A	1.825 (0.572)	1.685 (0.434)	−0.044 (0.052)	0.3999
LVIDs (cm)	3.400 (1.148)	3.571 (1.115)	0.128 (0.040)	0.0027	DT (msec)	134.50 (29.750)	119.860 (23.150)	−7.287 (3.186)	0.0267
LVPWd (cm)	0.660 (0.152)	0.586 (0.120)	−0.045 (0.013)	0.0008	Dtslope (m/s^2^)	6.214 (1.840)	7.185 (1.902)	0.642 (0.233)	0.0083
LVPWs(cm)	0.919 (0.159)	0.891 (0.158)	−0.017 (0.017)	0.3388	Sep E’ (cm/s)	8.911 (2.357)	8.783 (2.422)	−0.071 (0.171)	0.6788
EDV (mL)	104.285 (55.960)	93.633 (59.270)	−0.017 (0.017)	0.3388	Sep A’ (cm/s)	4.444 (1.267)	4.278 (1.549)	−0.259 (0.148)	0.0876
ESV (mL)	63.476 (45.000)	55.326 (45.750)	−8.749 (4.576)	0.0624	Sep S’ (cm/s)	5.023 (1.255)	5.389 (1.360)	0.114 (0.160)	0.4802
EF (%)	49.676 (15.344)	46.217 (14.120)	−2.680 (0.694)	0.0003	Sep E/E’	9.310 (2.483)	10.432 (4.032)	0.549 (0.300)	0.0737
SV (mL)	40.796 (13.436)	38.297 (16.540)	1.355 (2.472)	0.5866	Lat E’ (cm/s)	11.651 (3.800)	10.823 (3.689)	−0.420 (0.325)	0.2033
FS (%)	25.787 (8.635)	23.480 (8.000)	−1.722 (0.496)	0.0011	Lat A’(cm/s)	5.497 (1.604)	5.000 (1.765)	−0.413 (0.188)	0.0334
					Lat S’(cm/s)	4.283 (1.647)	5.333 (2.746)	0.369 (0.466)	0.4343
					Lat E/E’	6.987 (1.887)	8.401 (2.426)	0.565 (0.254)	0.0318

S.E: Standard error; IVSd: Interventricular septal end diastole; IVSs: Interventricular septal end systole; LVIDd: Left ventricular internal diameter end diastole; LVIDs: Left ventricular internal diameter end systole; LVPWd: Left ventricular posterior wall end diastole; LVPWs: Left ventricular posterior wall end systole; EDV: End-diastolic volume; ESV, End-systolic volume; EF: Ejection fraction; SV: Stroke volume; FS: Fractional shortening; DT: Deceleration time; E: peak early inflow velocity; A: peak late inflow velocity; Sep: septal; Lat: lateral, E’: peak early diastolic velocity; A’: peak late diastolic velocity; S’: peak systolic velocity.

**Table 3:** Linear regression analysis of the effect of cardiac medication and respiratory care on echocardiographic measurements.

	IVSd (cm)		IVSs (cm)		LVIDs (cm)	
	B (S.E)	*p*-Value	B (S.E)	*p*-Value	B (S.E)	*p*-Value
Beta-blocker	0.044 (0.039)	0.2696	0.021 (0.048)	0.6634	−0.029 (0.117)	0.3696
ACE inhibitor	0.012 (0.031)	0.7021	0.007 (0.038)	0.8646	−0.019 (0.094)	0.8443
Diuretic	−0.063 (0.026)	0.0213	−0.055 (0.033)	0.0951	0.108 (0.080)	0.1824
Aspirin	0.022 (0.028)	0.428	0.048 (0.033)	0.1605	−0.056 (0.082)	0.4996
Inotropic	0.018 (0.029)	0.543	0.051 (0.034)	0.1445	−0.032 (0.084)	0.7107
Ventilator	0.015 (0.028)	0.5849	0.013 (0.034)	0.7041	0.036 (0.082)	0.6631
Ventilator apply time per day (hour)	−0.001 (0.003)	0.7593	−0.001 (0.004)	0.8939	0.012 (0.009)	0.2057
	**LVPWd (cm)**		**EF (%)**		**FS (%)**	
	**B (S.E)**	***p*-Value**	**B (S.E)**	***p*-Value**	**B (S.E)**	***p*-Value**
Beta-blocker	0.033 (0.036)	0.3696	−3.483 (1.941)	0.0793	−0.266 (1.45)	0.8552
ACE inhibitor	−0.005 (0.029)	0.8572	−1.312 (1.596)	0.4154	−0.268 (1.095)	0.8079
Diuretic	−0.018 (0.025)	0.4877	−2.152 (1.358)	0.12	−1.368 (0.985)	0.1714
Aspirin	0.012 (0.026)	0.6512	0.071 (1.421)	0.9605	1.326 (0.985)	0.185
Inotropic	0.003 (0.026)	0.9241	−0.706 (1.439)	0.6259	0.239 (1.009)	0.814
Ventilator	−0.019 (0.025)	0.4505	−1.296 (1.399)	0.3592	−1.123 (0.984)	0.2584
Ventilator apply time per day (hour)	−0.001 (0.003)	0.6441	−0.087 (0.160)	0.5889	−0.097 (0.109)	0.3789
	**DT (ms)**		**DT slope (m/s^2^)**		**Lat A’ (cm/s)**	
	**B (S.E)**	***p*-Value**	**B (S.E)**	***p*-Value**	**B (S.E)**	***p*-Value**
Beta-blocker	1.720 (9.293)	0.854	0.374 (0.692)	0.5914	0.850 (0.550)	0.1297
ACE inhibitor	5.981 (7.322)	0.4182	−0.327 (0.535)	0.5436	−0.054 (0.401)	0.8929
Diuretic	−2.333 (6.535)	0.7227	−0.206 (0.461)	0.6564	−0.494 (0.367)	0.1855
Aspirin	−10.233 (6.233)	0.1075	0.405 (0.466)	0.3896	0.243 (0.383)	0.5293
Inotropic	−8.976 (6.559)	0.1778	−0.039 (0.481)	0.9361	0.579 (0.382)	0.1368
Ventilator	0.789 (6.472)	0.9035	−0.556 (0.461)	0.2348	−0.081 (0.381)	0.8337
Ventilator apply time per day (hour)	0.557 (0.736)	0.4533	−0.060 (0.053)	0.2692	−0.015 (0.044)	0.7329
	**Lat E/E’**					
	**B (S.E)**	***p*-Value**				
Beta-blocker	−0.138 (0.714)	0.8478				
ACE inhibitor	0.260 (0.537)	0.6308				
Diuretic	−0.204 (0.535)	0.7049				
Aspirin	0.282 (0.522)	0.5918				
Inotropic	0.074 (0.529)	0.8891				
Ventilator	0.400 (0.501)	0.4299				
Ventilator apply time per day (hour)	0.009 (0.059)	0.8791				

S.E: standard error; ACE inhibitor: angiotensin-converting enzyme inhibitors; IVSd: interventricular septal end diastole; IVSs: interventricular septal end systole; LVIDs: left ventricular internal diameter end systole; LVPWd: left ventricular posterior wall end diastole; EF: ejection fraction; FS: fractional shortening; DT: deceleration time; A’: peak late diastolic velocity; E: peak early inflow velocity; E’: peak early diastolic velocity.

**Table 4:** The change value of the ejection fraction between Group 1 and Group 2 with linear mixed model analysis.

Subgroup	EF (%)	EF2 (%)	B (Standard Error)	*p*-Value
Group 1	59.297 ± 8.407	53.594 ± 9.743	−3.615 (0.798)	0.0486
Group 2	33.923 ± 11.547	33.642 ± 9.736	−0.582 (1.266)	

Group 1: ejection fraction at initial treatment over 45%; Group 2: ejection fraction at initial treatment under 45%; EF: ejection fraction at the initial treatment; EF2: ejection fraction at the last treatment.

**Table 5:** Medication comparison between two groups with chi-square test.

Medication	Whether Taking	Total (*N* = 48)	Group 1 (*N* = 30)	Group 2 (*N* = 18)	*p*-Value
Beta-blocker	Yes	41	27	14	0.4002
	No	7	3	4	
ACE inhibitor	Yes	37	22	15	0.4991
	No	11	8	3	
Diuretic	Yes	26	14	12	0.1782
	No	22	16	6	
Aspirin	Yes	25	13	12	0.1172
	No	23	17	6	
Inotropic	Yes	28	12	16	0.0009
	No	20	18	2	

**Table 6:** Linear mixed model analysis of the effect of cardiac medication and respiratory care on echocardiographic measurements in each group.

	IVSd (cm) in Group 1		IVSd (cm) in Group 2		*p*-Value *	IVSs (cm) in Group 1		IVSs (cm) in Group 2		*p*-Value *
	B (S.E)	*p*-Value	B (S.E)	*p*-Value		B (S.E)	*p*-Value	B (S.E)	*p*-Value	
Beta-blocker	0.052 (0.043)	0.2415	0.027 (0.067)	0.6888	0.9102	0.039 (0.058)	0.5047	−0.035 (0.080)	0.6786	0.9102
ACE inhibitor	−0.001 (0.030)	0.9616	0.191 (0.059)	0.0039	0.0408	−0.026 (0.030)	0.5196	0.239 (0.088)	0.0127	0.0408
Diuretic	−0.055 (0.020)	0.0541	−0.115 (0.090)	0.2295	0.4655	−0.069 (0.030)	0.0654	0.040 (0.116)	0.7351	0.4655
Aspirin	0.023 (0.028)	0.4142	0.006 (0.063)	0.9254	0.8844	0.074 (0.034)	0.0384	0.018 (0.076)	0.8178	0.8844
Inotropic	0.023 (0.027)	0.4009	0.136 (0.094)	0.1635	0.3341	0.072 (0.033)	0.0361	−0.015 (0.130)	0.9092	0.3341
Ventilator	0.008 (0.029)	0.7954	−0.017 (0.050)	0.7515	0.6758	0.017 (0.038)	0.6574	−0.070 (0.063)	0.2769	0.6758
	**LVIDd (cm) in Group 1**		**LVIDd (cm) in Group 2**		*p*-Value *	**LVIDs (cm) in Group 1**		**LVIDs (cm) in Group 2**		*p*-Value *
	**B (S.E)**	***p*-Value**	**B (S.E)**	***p*-Value**		**B (S.E)**	***p*-Value**	**B (S.E)**	***p*-Value**	
Beta-blocker	−0.162 (0.160)	0.3216	−0.384 (0.260)	0.1550	0.5472	−0.129 (0.140)	0.3755	−0.042 (0.240)	0.8663	0.6953
ACE inhibitor	−0.107 (0.100)	0.3284	−0.219 (0.340)	0.5380	0.5986	−0.093 (0.097)	0.3476	−0.168 (0.310)	0.5938	0.5845
Diuretic	0.107 (0.105)	0.3168	0.512 (0.408)	0.2233	0.47944	0.120 (0.092)	0.2003	0.705 (0.382)	0.0788	0.3563
Aspirin	0.086 (0.105)	0.4167	−0.153 (0.230)	0.5265	0.4814	0.106 (0.093)	0.2637	−0.001 (0.226)	0.9991	0.6239
Inotropic	−0.039 (0.100)	0.7018	−0.513 (0.460)	0.2801	0.2265	−0.031 (0.080)	0.7260	−0.270 (0.422)	0.5283	0.3862
Ventilator	−0.062 (0.100)	0.5681	−0.131 (0.220)	0.5715	0.6733	0.008 (0.095)	0.9353	−0.188 (0.200)	0.3765	0.3211
	**LVPWd (cm) in Group 1**		**LVPWd (cm) in Group 2**		*p*-Value *	**LVPWs (cm) in Group 1**		**LVPWs (cm) in Group 2**		*p*-Value *
	**B (S.E)**	***p*-Value**	**B (S.E)**	***p*-Value**		**B (S.E)**	***p*-Value**	**B (S.E)**	***p*-Value**	
Beta-blocker	0.060 (0.048)	0.2199	−0.040 (0.059)	0.5033	0.3869	0.034 (0.069)	0.6306	0.008 (0.042)	0.8468	0.9761
ACE inhibitor	−0.023 (0.030)	0.4828	0.129 (0.058)	0.0378	0.036	−0.017 (0.040)	0.7084	0.033 (0.055)	0.5526	0.5405
Diuretic	−0.018 (0.030)	0.5703	−0.012 (0.070)	0.8788	0.8898	−0.024 (0.040)	0.5986	−0.121 (0.060)	0.0576	0.7605
Aspirin	0.004 (0.031)	0.8876	−0.022 (0.050)	0.6709	0.8216	−0.009 (0.040)	0.8440	−0.220 (0.040)	0.5888	0.8872
Inotropic	−0.006 (0.030)	0.8313	0.081 (0.086)	0.3590	0.2695	−0.049 (0.040)	0.2402	0.043 (0.066)	0.5252	0.4079
Ventilator	−0.018 (0.030)	0.5751	−0.014 (0.040)	0.7467	0.9996	−0.036 (0.040)	0.4186	0.028 (0.039)	0.4879	0.3906
	**EDV (mL) in Group 1**		**EDV (mL) in Group 2**		*p*-Value *	**ESV (mL) in Group 1**		**ESV (mL) in Group 2**		*p*-Value *
	**B (S.E)**	***p*-Value**	**B (S.E)**	***p*-Value**		**B (S.E)**	***p*-Value**	**B (S.E)**	***p*-Value**	
Beta-blocker	−42.170 (27.500)	0.1369	−61.310 (24.200)	0.0215	0.4629	−21.263 (16.700)	0.2141	−28.740 (21.500)	0.1995	0.7485
ACE inhibitor	−19.070 (12.100)	0.1280	−40.350 (31.300)	0.2146	0.6434	−11.580 (6.450)	0.0837	−36.170 (23.100)	0.1363	0.3155
Diuretic	13.180 (11.260)	0.2518	1.605 (30.909)	0.9592	0.4663	8.648 (5.997)	0.1608	6.707 (24.460)	0.7873	0.4025
Aspirin	2.255 (10.940)	0.8383	−23.740 (22.520)	0.3064	0.5379	4.827 (5.882)	0.4191	−4.860 (20.400)	0.8146	0.8402
Inotropic	−13.130 (9.160)	0.1631	−85.240 (45.610)	0.0790	0.0813	−6.720 (4.905)	0.1820	−665.800 (36.400)	0.0887	0.0554
Ventilator	1.167 (12.230)	0.9247	20.963 (23.580)	0.3865	0.0898	2.157 (6.700)	0.7499	25.950 (16.960)	0.1443	0.0100
	**EF (%) in Group 1**		**EF (%) in Group 2**		*p*-Value *	**SV (mL) in Group 1**		**SV (mL) in Group 2**		*p*-Value *
	**B (S.E)**	***p*-Value**	**B (S.E)**	***p*-Value**		**B (S.E)**	***p*-Value**	**B (S.E)**	***p*-Value**	
Beta-blocker	0.276 (2.549)	0.9144	−7.623 (2.700)	0.0099	0.2237	−20.260 (12.120)	0.1062	−42.860 (6.470)	<0.0001	0.0698
ACE inhibitor	0.019 (1.707)	0.9912	0.152 (4.070)	0.9706	0.7009	−7.705 (6.000)	0.2100	−1.719 (22.800)	0.9409	0.2604
Diuretic	−0.616 (1.650)	0.7120	−2.774 (4.590)	0.5525	0.6105	3.860 (5.584)	0.4953	−4.708 (11.000)	0.675	0.2091
Aspirin	−2.350 (1.622)	0.1556	−4.186 (2.870)	0.1588	0.6596	−3.471 (5.320)	0.5201	−22.220 (6.940)	0.0053	0.0108
Inotropic	0.171 (1.528)	0.9113	−4.287 (5.233)	0.4215	0.7733	−6.525 (4.630)	0.1709	−17.160 (33.400)	0.6145	0.8761
Ventilator	−0.875 (1.590)	0.5861	4.269 (2.675)	0.1247	0.1180	−1.64 (5.692)	0.7752	−13.490 (14.000)	0.3507	0.4352
	**FS (%) in Group 1**		**FS (%) in Group 2**		*p*-Value *	**Mitral E (m/s) in Group 1**		**Mitral E (m/s) in Group 2**		*p*-Value *
	**B (S.E)**	***p*-Value**	**B (S.E)**	***p*-Value**		**B (S.E)**	***p*-Value**	**B (S.E)**	***p*-Value**	
Beta-blocker	−0.078 (1.870)	0.9672	−3.005 (1.310)	0.0327	0.6099	5.015 (4.460)	0.2692	4.036 (5.141)	0.4412	0.4521
ACE inhibitor	0.223 (1.332)	0.8681	−0.001 (1.600)	0.9994	0.8995	−0.032 (3.140)	0.992	−3.590 (6.685)	0.5969	0.6398
Diuretic	−0.911 (1.260)	0.4766	−1.529 (1.970)	0.4475	0.8784	−2.417 (3.050)	0.4339	0.025 (7.997)	0.9975	0.9696
Aspirin	−1.430 (1.270)	0.2682	−0.122 (1.420)	0.9326	0.9436	−3.073 (2.967)	0.3078	4.849 (4.763)	0.3202	0.2114
Inotropic	0.222 (1.233)	0.8584	−2.612 (1.927)	0.1892	0.8177	−5.527 (2.750)	0.0534	−0.519 (9.440)	0.9567	0.585
Ventilator	−0.163 (1.250)	0.8970	2.180 (1.140)	0.0689	0.2167	−3.903 (2.915)	0.1898	9.385 (4.194)	0.0362	0.0296
	**Mitral A (cm/s) in Group 1**		**Mitral A (cm/s) in Group 2**		*p*-Value *	**E/A in Group 1**		**E/A in Group 2**		*p*-Value *
	**B (S.E)**	***p*-Value**	**B (S.E)**	***p*-Value**		**B (S.E)**	***p*-Value**	**B (S.E)**	***p*-Value**	
Beta-blocker	−2.774 (4.940)	0.5787	1.937 (8.074)	0.8127	0.5846	0.142 (0.155)	0.3648	−0.543 (0.380)	0.1704	0.0635
ACE inhibitor	−4.999 (3.090)	0.1161	−12.750 (10.230)	0.2264	0.3582	0.160 (0.103)	0.1288	0.292 (0.459)	0.5315	0.6024
Diuretic	−3.093 (3.210)	0.3433	−11.560 (9.149)	0.2199	0.0809	0.093 (0.105)	0.3827	0.548 (0.388)	0.1720	0.187
Aspirin	−2.403 (3.070)	0.4401	5.676 (6.951)	0.4234	0.1879	−0.052 (0.104)	0.6208	−0.464 (0.280)	0.1183	0.0807
Inotropic	−2.625 (3.000)	0.3880	−12.050 (14.664)	0.4205	0.4166	−0.076 (0.103)	0.4693	0.380 (0.634)	0.5558	0.2528
Ventilator	−2.603 (3.070)	0.4034	8.671 (6.312)	0.1840	0.0602	−0.029 (0.105)	0.7821	−0.224 (0.270)	0.4264	0.5208
	**DT (msec) in Group 1**		**DT (msec) in Group 2**		*p*-Value *	**Dtslope (m/s^2^) in Group 1**		**Dtslope (m/s^2^) in Group 2**		*p*-Value *
	**B (S.E)**	***p*-Value**	**B (S.E)**	***p*-Value**		**B (S.E)**	***p*-Value**	**B (S.E)**	***p*-Value**	
Beta-blocker	5.760 (11.040)	0.6055	−0.085 (15.000)	0.9955	0.9809	0.217 (0.754)	0.7748	0.401 (1.13)	0.7260	0.7318
ACE inhibitor	7.043 (7.366)	0.3459	5.351 (21.630)	0.8070	0.9029	−0.405 (0.504)	0.4275	−1.493 (1.670)	0.3838	0.8565
Diuretic	−6.826 (7.220)	0.3515	19.768 (18.900)	0.3075	0.3474	−0.027 (0.509)	0.9587	−0.175 (1.250)	0.8910	0.8608
Aspirin	−12.450 (6.540)	0.0659	0.072 (13.060)	0.9956	0.3720	0.081 (0.500)	0.8724	0.984 (0.970)	0.3222	0.4438
Inotropic	−14.900 (6.500)	0.0285	31.220 (29.582)	0.3032	0.1105	0.172 (0.489)	0.7274	−0.893 (2.060)	0.6692	0.6133
Ventilator	−5.863 (7.190)	0.4209	15.510 (12.320)	0.2219	0.1295	−0.494 (0.488)	0.3196	−0.511 (0.870)	0.5647	0.8518
	**Sep E’ (cm/s) in Group 1**		**Sep E’ (cm/s) in Group 2**		*p*-Value *	**Sep A’ (cm/s) in Group 1**		**Sep A’ (cm/s) in Group 2**		*p*-Value *
	**B (S.E)**	***p*-Value**	**B (S.E)**	***p*-Value**		**B (S.E)**	***p*-Value**	**B (S.E)**	***p*-Value**	
Beta-blocker	−0.214 (0.630)	0.7380	−0.339 (1.040)	0.7496	0.8386	−0.274 (0.640)	0.671	0.413 (0.589)	0.4906	0.5893
ACE inhibitor	−0.444 (0.400)	0.2838	0.443 (1.308)	0.7384	0.4364	−0.010 (0.432)	0.9819	1.580 (0.737)	0.0439	0.0692
Diuretic	0.569 (0.401)	0.1649	0.881 (1.480)	0.5579	0.9368	−0.118 (0.421)	0.7812	−1.271 (0.760)	0.1099	0.2247
Aspirin	0.559 (0.379)	0.1503	−0.807 (0.892)	0.3762	0.0976	−0.133 (0.389)	0.7352	0.157 (0.528)	0.7694	0.9436
Inotropic	−0.069 (0.390)	0.8634	3.295 (1.509)	0.0405	0.0172	−0.067 (0.397)	0.8667	2.192 (0.963)	0.0335	0.0595
Ventilator	−0.153 (0.400)	0.7049	0.626 (0.857)	0.4733	0.5496	−0.026 (0.407)	0.9500	−0.042 (0.490)	0.9334	0.7168
	**Sep S’ (cm/s) in Group 1**		**Sep S’ (cm/s) in Group 2**		*p*-Value *	**Sep E/E’ in Group 1**		**Sep E/E’ in Group 2**		*p*-Value *
	**B (S.E)**	***p*-Value**	**B (S.E)**	***p*-Value**		**B (S.E)**	***p*-Value**	**B (S.E)**	***p*-Value**	
Beta-blocker	0.709 (0.523)	0.1841	0.315 (0.737)	0.6736	0.6996	0.984 (0.849)	0.2545	0.578 (1.780)	0.7486	0.5182
ACE inhibitor	0.222 (0.364)	0.5463	−2.146 (1.17)	0.0824	0.0128	0.611 (0.595)	0.3126	−0.924 (2.180)	0.6767	0.4498
Diuretic	0.658 (0.329)	0.0538	0.621 (0.627)	0.3333	0.9380	−0.836 (0.579)	0.1579	−6.780 (2.101)	0.004	0.3941
Aspirin	−0.334 (0.330)	0.3228	1.025 (0.536)	0.0696	0.2263	−0.892 (0.560)	0.1225	0.963 (1.537)	0.5378	0.2673
Inotropic	−0.423 (0.320)	0.2075	0.988 (1.875)	0.6040	0.3102	−0.604 (0.590)	0.3156	−3.008 (2.690)	0.2768	0.3497
Ventilator	0.018 (0.356)	0.9591	0.452 (0.639)	0.4868	0.3152	−0.273 (0.590)	0.6472	1.803 (1.471)	0.2339	0.0557
	**Lat E’ (cm/s) in Group 1**		**Lat E’ (cm/s) in Group 2**		*p*-Value *	**Lat A’ (cm/s) in Group 1**		**Lat A’ (cm/s) in Group 2**		*p*-Value *
	**B (S.E)**	***p*-Value**	**B (S.E)**	***p*-Value**		**B (S.E)**	***p*-Value**	**B (S.E)**	***p*-Value**	
Beta-blocker	0.405 (1.240)	0.7465	1.465 (1.809)	0.4287	0.8387	0.572 (0.646)	0.3831	2.648 (1.345)	0.0655	0.2615
ACE inhibitor	−0.747 (0.850)	0.3891	1.678 (1.531)	0.2876	0.1776	−0.028 (0.445)	0.9505	0.514 (1.193)	0.6718	0.7572
Diuretic	−0.552 (0.860)	0.5285	1.020 (2.413)	0.6774	0.2243	−0.369 (0.443)	0.4124	−0.839 (1.290)	0.5251	0.2938
Aspirin	0.022 (0.888)	0.9808	−0.915 (1.528)	0.5568	0.9290	−0.033 (0.941)	0.9407	0.777 (1.018)	0.4554	0.6567
Inotropic	−0.933 (0.850)	0.285	3.028 (1.789)	0.1077	0.0797	0.416 (0.447)	0.3597	3.368 (1.324)	0.0210	0.0533
Ventilator	−0.413 (0.860)	0.6359	0.071 (1.340)	0.9585	0.8334	0.166 (0.439)	0.6778	−0.223 (0.260)	0.4023	0.3666
	**Lat S’ (cm/s) in Group 1**		**Lat S’ (cm/s) in Group 2**		*p*-Value *	**Lat E/E’ in Group 1**		**Lat E/E’ in Group 2**		*p*-Value *
	**B (S.E)**	***p*-Value**	**B (S.E)**	***p*-Value**		**B (S.E)**	***p*-Value**	**B (S.E)**	***p*-Value**	
Beta-blocker						−0.466 (0.967)	0.6335	−1.440 (1.313)	0.2881	0.6480
ACE inhibitor						0.681 (0.646)	0.3006	−2.751 (1.070)	0.0205	0.009
Diuretic						0.284 (0.692)	0.6841	−1.581 (1.500)	0.3090	0.0884
Aspirin			2.906 (1.155)	0.0258		0.069 (0.687)	0.9204	0.899 (1.124)	0.4350	0.4729
Inotropic	0.504 (0.472)	0.2973	−4.103 (1.982)	0.0589	0.0495	0.229 (0.689)	0.7418	−3.390 (1.673)	0.0779	0.0756
Ventilator	1.152 (0.324)	0.0017	−0.447 (1.455)	0.7636	0.6628	0.096 (0.691)	0.8900	0.731 (0.981)	0.4666	0.5588

S.E: standard error, ACE inhibitor: angiotensin-converting enzyme inhibitors, IVSd: interventricular septal end diastole, IVSs: interventricular septal end systole, LVIDd: left ventricular internal diameter end diastole, LVIDs: left ventricular internal diameter end systole, LVPWd: left ventricular posterior wall end diastole, LVPWs: left ventricular posterior wall end systole, EDV: end-diastolic volume, ESV: end-systolic volume, EF: ejection fraction, SV: stroke volume, FS: fractional shortening, DT: deceleration time, E: peak early inflow velocity, A: peak late inflow velocity, E’: peak early diastolic velocity, A’: peak late diastolic velocity, S’: peak systolic velocity. *p*-value *; comparison between two groups’ change values of echocardiographic data according to cardiac medications or respiratory care.

## References

[1] Deconinck N., Dan B. (2007). Pathophysiology of Duchenne Muscular Dystrophy: Current Hypotheses. Pediatric Neurol..

[2] Bushby K., Finkel R., Birnkrant D.J., Case L.E., Clemens P.R., Cripe L., Kaul A., Kinnett K., McDonald C., Pandya S. (2010). Diagnosis and management of Duchenne muscular dystrophy, part 1: Diagnosis, and pharmacological and psychosocial management. Lancet Neurol..

[3] Eagle M., Baudouin S.V., Chandler C., Giddings D.R., Bullock R., Bushby K. (2002). Survival in Duchenne muscular dystrophy: Improvements in life expectancy since 1967 and the impact of home nocturnal ventilation. Neuromuscul. Disord..

[4] Gomez-Merino E., Bach J.R. (2002). Duchenne Muscular Dystrophy. Am. J. Phys. Med. Rehabil..

[5] Finder J.D., Birnkrant D., Carl J., Farber H.J., Gozal D., Iannaccone S.T., Kovesi T., Kravitz R.M., Panitch H., Schramm C. (2004). Respiratory care of the patient with Duchenne muscular dystrophy: ATS consensus statement. Am. J. Respir. Crit. Care Med..

[6] El-Aloul B., Altamirano-Diaz L., Zapata-Aldana E., Rodrigues R., Malvankar-Mehta M.S., Nguyen C.T. (2017). Pharmacological therapy for the prevention and management of cardiomyopathy in Duchenne muscular dystrophy: A systematic review. Neuromuscul. Disord..

[7] Kwon S.W., Kang S.W., Kim J.Y., Choi E.Y., Yoon Y.W., Park Y.M., Ma D.W., Chung H., Kwon H.M., Rim S.J. (2012). Outcomes of cardiac involvement in patients with late-stage Duchenne muscular dystrophy under management in the pulmonary rehabilitation center of a tertiary referral hospital. Cardiology.

[8] Raman S.V., Hor K.N., Mazur W., Halnon N.J., Kissel J.T., He X., Tran T., Smart S., McCarthy B., Taylor M.D. (2015). Eplerenone for early cardiomyopathy in Duchenne muscular dystrophy: A randomised, double-blind, placebo-controlled trial. Lancet Neurol..

[9] Stein C.A. (2016). Eteplirsen approved for Duchenne muscular dystrophy: The FDA faces a difficult choice. Mol. Ther..

[10] Viollet L., Thrush P.T., Flanigan K.M., Mendell J.R., Allen H.D. (2012). Effects of angiotensin-converting enzyme inhibitors and/or beta blockers on the cardiomyopathy in Duchenne muscular dystrophy. Am. J. Cardiol..

[11] Duboc D., Meune C., Pierre B., Wahbi K., Eymard B., Toutain A., Berard C., Vaksmann G., Weber S., Bécane H.M. (2007). Perindopril preventive treatment on mortality in Duchenne muscular dystrophy: 10 years’ follow-up. Am. Heart J..

[12] Cox G.F., Kunkel L.M. (1997). Dystrophies and heart disease. Curr. Opin. Cardiol..

[13] Lee S., Lee H., Eun L.Y., Gang S.W. (2018). Cardiac function associated with home ventilator care in Duchenne muscular dystrophy. Korean J. Pediatrics.

[14] Barber B.J., Andrews J.G., Lu Z., West N.A., Meaney F.J., Price E.T., Gray A., Sheehan D.W., Pandya S., Yang M. (2013). Oral corticosteroids and onset of cardiomyopathy in Duchenne muscular dystrophy. J. Pediatrics.

[15] Lai W.W., Geva T., Shirali G.S., Frommelt P.C., Humes R.A., Brook M.M., Pignatelli R.H., Rychik J. (2006). Guidelines and standards for performance of a pediatric echocardiogram: A report from the Task Force of the Pediatric Council of the American Society of Echocardiography. J. Am. Soc. Echocardiogr..

[16] Klitzner T.S., Beekman R.H., Galioto F.M., Jones T.K., Manning P., Morrow W.R., Colegrove L. (2005). Cardiovascular health supervision for individuals affected by Duchenne or Becker muscular dystrophy. Pediatrics.

[17] Goudot F.-X., Wahbi K., Aïssou L., Sorbets E., Siam-Tsieu V., Eymard B., Themar Noel C., Devaux J.Y., Dessault O., Duboc D. (2015). Reduced inotropic reserve is predictive of further degradation in left ventricular ejection fraction in patients with Duchenne muscular dystrophy. Eur. J. Heart Fail..

[18] Birnkrant D.J., Bushby K., Bann C.M., Apkon S.D., Blackwell A., Brumbaugh D., Case L.E., Clemens P.R., Hadjiyannakis S., Pandya S. (2018). Diagnosis and management of Duchenne muscular dystrophy, part 1: Diagnosis, and neuromuscular, rehabilitation, endocrine, and gastrointestinal and nutritional management. Lancet Neurol..

[19] Lam C.S., Solomon S.D. (2014). The middle child in heart failure: Heart failure with mid-range ejection fraction (40–50%). Eur. J. Heart Fail..

[20] Giatrakos N., Kinali M., Stephens D., Dawson D., Muntoni F., Nihoyannopoulos P. (2006). Cardiac tissue velocities and strain rate in the early detection of myocardial dysfunction of asymptomatic boys with Duchenne’s muscular dystrophy: Relationship to clinical outcome. Heart.

[21] Gloss D., Moxley R.T., Ashwal S., Oskoui M. (2016). Practice guideline update summary: Corticosteroid treatment of Duchenne muscular dystrophy: Report of the Guideline Development Subcommittee of the American Academy of Neurology. Neurology.

